# On Filling Teeth, after the Pulp Has Suppurated, or Where It Has Been Destroyed with a View to This Operation

**Published:** 1850-04

**Authors:** Sam'l Rambo

**Affiliations:** Montgomery, Al'a.


### ARTICLE II.

*On Filling Teeth, after the Pulp has Suppurated, or where it has been Destroyed with a View to this Operation.* By SAM'L RAMBO, M. D., of Montgomery, Al'a.

PROF'R C. A. HARRIS, *Dear Sir:*

"Situated as I am," with a mixed practice from town and country, patients sometimes present themselves that *cannot* undergo the necessary medical treatment, laid down by authorities, preparatory to the operation of plugging. Many come from a distance, and, having no idea whatever of what is really necessary, insist on an operation being performed *immediately*, though a nerve may be exposed and suppurating, or an abscess already formed in the gum and alveoli.

Some, with teeth thus situated, *decline* extraction, from "certain states of constitutional health, and the fears of timidity of the patient," added to what they conceive to be the proper operation to be done. They are averse to extraction, or any mode of treatment that would require more than an hour, and plead distance, inconvenience, and what *they* "think about it." Science "goes to the wall," if the usual operation of plugging is performed, and an abscess, with its influences, *established*, as a "fixed fact."

I am sometimes consulted by patients who are threatened with alveolar abscess, from the suppuration of the nerve-pulp, after the tooth has been filled, and when the crown is still strong, well-formed, and of good color. In such cases I sometimes think it advisable to attempt the preservation of the tooth, and more particularly when the patient is opposed to extraction, or a regular course of medical treatment of the nerve, as proposed by yourself, and Drs. Maynard and Baker.

Under these circumstances, I have a compromise operation, which the above necessities have induced me to adopt, and although it may be known to many operators, yet it is comparatively new practice with me, not having performed it until within two or three years past.

The operation I propose, I think advisable, where a radical cure of abscess cannot be effected by excavating the fang, and the use of injections, or where there is any uncertainty in regard to the condition of the sac.

It is very difficult to ensure a cure under the most favorable circumstances, and cases do occur where the aperture through the apex of the fang is so small, that injections cannot pass in quantity sufficient to produce the desired result.

I have no doubt but that the practice of filling the fang, is founded on correct views, when an abscess has never existed, and when the nerve and alveolar-dental-tissues have not suffered from the effects of arsenic, or other agents used for the destruction of the nerve within the root, and where a healthy cicatrix of the nerve has formed. If there is no inflammation or secretion, there can be no abscess, and I cannot imagine how the filling of a tooth in this condition, could produce it. But my practice applies to cases where there is danger of an abscess forming at the apex of the fangs, in teeth that have been filled, and have subsequently lost their vitality, or in cases of abscess from any other cause, where the periosteum of the root has been but little injured, where the patient refuses to have the nerve cavity treated, or where, from the nature of the case, delay is unnecessary.

The following is my plan of procedure, viz: The gum of the affected tooth may, or may not, be split, as circumstances may require, in order to pass a small drill under the edge of the gum into the nerve canal, directing the drill a little upward towards the apex of the root; when the nerve cavity has been reached, introduce a small, flexible wire up to the apex, if possible; pressure should then be made over the region of the abscess, or swollen point, and the pus forced through the nerve-canal from the sac, and discharged through the drilled aperture under the edge of the gum. The old fillings, if any, should next be removed; the nerve-cavity freely opened, and the remains of the nerve, and discolored bone, if any, removed. The canal to the apex of the root, should be thoroughly cleansed, and injections used of water, and sol. nit. aegt., or other astringents, forcing them through into the sac, if possible. The external abscess should then be opened, and injections thrown in; after this treatment, the tooth is ready for the filling.

A piece of strong wire, the size of the drilled aperture, should now be introduced through the same, far enough to prevent any gold foil from passing beyond it, and protruding a little; it serves, also, as a point of resistance for the consolidation of the first portion of the plug. The plug is then finished in the usual way, and the wire, (which should be of gold,) withdrawn by plyers or other instruments.

After the operation, the pus that may form in the sac, finds a new channel of escape, and decreases in quantity, as the sac, from not being distended by accumulation, decreases in size; the external abscess heals, the adjacent tissues become more consolidated and healthy, and future abscess avoided.

The gum, acting like a valve, allows the secretions to escape, but prevents the entrance of matter from without. Finally, the pus becomes almost imperceptible in quantity, from the contraction of the sac, and the tooth answers about as good a purpose as ordinary pivot teeth, but preferable, from the fact that the natural crown is preserved.

Have you any experience with this plan of treatment? If so, I should be pleased to hear your views on the subject.

I am yours, very truly,

SAM'L RAMBO.

Montgomery, *Feb.* 1*st*, 1850.

